# The Terms of “You(s)”: How the Term of Address Used by Conversational Agents Influences User Evaluations in French and German Linguaculture

**DOI:** 10.3389/fpubh.2021.691595

**Published:** 2022-01-05

**Authors:** Joseph Ollier, Marcia Nißen, Florian von Wangenheim

**Affiliations:** ^1^Chair of Technology Marketing, Department of Management, Economics and Technology (D-MTEC), ETH Zürich, Zurich, Switzerland; ^2^Centre for Digital Health Interventions (CDHI), Department of Management, Economics and Technology (D-MTEC), ETH Zürich, Zurich, Switzerland

**Keywords:** conversational agents, chatbots, term of address, T/V distinction, linguaculture, digital health

## Abstract

**Background:** Conversational agents (CAs) are a novel approach to delivering digital health interventions. In human interactions, terms of address often change depending on the context or relationship between interlocutors. In many languages, this encompasses *T/V distinction*—formal and informal forms of the second-person pronoun “You”—that conveys different levels of familiarity. Yet, few research articles have examined whether CAs' use of T/V distinction across language contexts affects users' evaluations of digital health applications.

**Methods:** In an online experiment (*N* = 284), we manipulated a public health CA prototype to use either informal or formal T/V distinction forms in French (“tu” vs. “vous”) and German (“du” vs. “Sie”) language settings. A MANCOVA and *post-hoc* tests were performed to examine the effects of the independent variables (i.e., T/V distinction and Language) and the moderating role of users' demographic profile (i.e., Age and Gender) on eleven user evaluation variables. These were related to four themes: (i) Sociability, (ii) CA-User Collaboration, (iii) Service Evaluation, and (iv) Behavioral Intentions.

**Results:** Results showed a four-way interaction between T/V Distinction, Language, Age, and Gender, influencing user evaluations across all outcome themes. For French speakers, when the informal “T form” (“*Tu”*) was used, higher user evaluation scores were generated for younger women and older men (e.g., the CA felt more humanlike or individuals were more likely to recommend the CA), whereas when the formal “V form” (“*Vous”*) was used, higher user evaluation scores were generated for younger men and older women. For German speakers, when the informal T form (“*Du”*) was used, younger users' evaluations were comparable regardless of Gender, however, as individuals' Age increased, the use of “*Du”* resulted in lower user evaluation scores, with this effect more pronounced in men. When using the formal V form (“*Sie”*), user evaluation scores were relatively stable, regardless of Gender, and only increasing slightly with Age.

**Conclusions:** Results highlight how user CA evaluations vary based on the T/V distinction used and language setting, however, that even within a culturally homogenous language group, evaluations vary based on user demographics, thus highlighting the importance of personalizing CA language.

## Introduction

### Designing Conversational Agents for Healthcare

Conversational agents (CAs) are intelligent computer programs that engage users in human-like conversations and include text-based chatbots, voice-activated assistants, and embodied conversational agents (1). The use of CAs in healthcare service delivery has become increasingly widespread as organizations and practitioners recognize their ability to transform the healthcare sector and empower individuals to co-manage their care effectively (2). A broad range of scientifically evaluated healthcare chatbots are currently (commercially) available, providing digital health solutions across the patient journey from diagnostic conversations of a regular doctor visit [e.g., Babylon^1^ and Ada^2^ (3)], consultations on sensitive health topics (e.g., the Hiv Chatbot^3^)., therapy for specific chronic diseases [e.g., mental health (4): WOEBOT^4^ (5, 6), WYSA^5^ (7), and TESS^6^ (8–10); cardiovascular diseases: FLORENCE^7^ (11)] to general lifestyle health [e.g., LARK^8^ (12)]. Benefits of CA use in healthcare include improving availability, personalization, and efficacy of service delivery (13). Moreover, due to their highly scalable nature, CAs have been noted as a promising method to address health disparities between developed and developing nations and ensure equitable healthcare service delivery worldwide (14).

From initial investigations into the suitability of CAs to act as healthcare partners (15), research has now turned to understanding best design practices for the anthropomorphic user interfaces that CAs use (16, 17). Research has demonstrated how visual, conversational, and identity-related cues trigger “humanness heuristics” (18, 19) and affective states in users similar to natural human communication (20). Design factors such as physical appearance (21), gender (22, 23), and speech dialect (24) can be tailored to match users' cultural and demographic background and help to establish rapport (15, 20) and perceptions of a CA's personality (25). Language-based cues are of particular importance due to their strong role in driving user engagement (1). For example, research has highlighted how CA use of task and social-based communication (15, 26, 27), politeness (28), interactivity (17, 18), and information quality (29) have been linked to user evaluation outcomes such as interpersonal closeness (22), intention to use (30), satisfaction (31, 32), trust (33), and user self-disclosure (34).

To date though, few research articles have examined important cultural- and sociolinguistic phenomena in CA design across diverse *linguacultures* (i.e., where language and culture constitute a single domain), and how these influence perceptions of CAs and their effectiveness in healthcare service delivery (14). This is particularly important, however, as language has a strong impact on social cognition and the co-construction of meaning between dyadic conversational partners (35, 36). For example, in English-speaking contexts, *terms of address* such as “Sir” (37) or “Mate” (38) vary in contextual appropriateness depending on the focus of address (e.g., police officer, friend). In CA contexts using users' first names as the term of address has been linked to increased perceptions of CA politeness and thoughtfulness, with the caveat that this may be bound to cultural limits and preferences (25). In scaling up digital-health interventions globally, it is therefore imperative to further investigate CA language phenomena such as the term of address in diverse language contexts (14, 39).

### Term of Address: Design Considerations

In the current study, we examine a particular term of address cue: *T/V distinction* or *Tu/Vous distinction* (40), which refers to the use of different second-person pronouns (“You”) in some languages, denoting a combination of less (T form) or more (V form) formality, distance, or emotional detachment (41). Arising originally from the Latin pronouns “*Tu”* and “*Vos”* (becoming “Thou” and “You” in English), T/V distinction is widely used in Indo-European languages (42), for example, “*du”* and “*Sie”* in German, “*tu”* and “*vous”* in French, “*tú”* and “*vosotros/vosotras/usted(es)”* in Spanish, with similar use in other non-Latin related languages such as Chinese, Malaysian, and Korean (43). T/V distinction is said to encode interactional meanings and shape normative expectations (44), such as politeness etiquette (41, 45, 46), which when breached by a communication partner may disrupt the cultural script in play (44), and be perceived as an insult (41), membership of a different social class (47), affiliation with another culture or grouping (48), and lead to outcomes such as customer dissatisfaction (43, 49, 50).

For designers of CAs, therefore, it remains vital to investigate CA T/V distinction usage to facilitate engaging user experiences (51). As individuals look for cues in the cultural script to orient themselves and understand potential outcomes, benefits, or goals of relationships (52), designers of CAs can provide clear and stable meaning by appropriate utilization of the T/V form for a given user group (45). In a wider public health context, ensuring the correct reception of CA-based technology can help extend healthcare service equitably across the world, and address some of the shortages of human resources for health and clinical services, to “improve accessibility, availability, affordability, and acceptability of public health services worldwide” (20). Yet, as highlighted by the World Health Organization, to do so, the research community must address the risk of design biases when developing new interventions for diverse cultural backgrounds (14) which when simply transferred from English-speaking contexts may apply a “cultural filtering” effect (43) and disregard important communicative nuances in cultural scripts of a given linguaculture (43, 49).

The current research, therefore, investigates CA use of T/V distinction in two unique linguacultures of French and German and explores the role T/V distinction plays in user evaluation of eleven outcome variables grouped into the following themes: (i) Sociability (i.e., Social Presence, and Conversational Enjoyment), (ii) CA-User Collaboration (i.e., CA Trust, Co-Production, Perceived Privacy Protection, and Privacy Concern), (iii) Service Evaluation (i.e., Perceived Ease of Use, Perceived Usefulness, and Service Satisfaction) and (iv) Behavioral Intentions (i.e., Intended Usage, and Net Promoter Score). Further information on these outcome variables is available in the Supplementary Materials.

### Hypotheses Development

Similarities and variations in the usage of T/V distinction between linguacultures have been demonstrated with regard to contextual appropriateness (45), the relationship between interlocutors (52), and subtleties in semantic meanings (43, 49). For French speakers, utilization of V form generally occurs more frequently in interactions as a method to convey a base level of respect for all including strangers (44, 53) as well as to exhibit respect to hierarchy (54). In German, V form is employed less frequently, with many forms of relationships progressing to T form immediately or after a short interaction (44). While T form occurs more frequently in German, its usage however typically offers less significance as a relationship marker, being more readily extended to acquaintances or strangers (44), and lacking as rich connotations of proximity, intimacy, and positive affect as in French-speaking settings (55). Nevertheless, contextual influences remain highly consequential (45), and in certain German-speaking settings, such as when dealing with customer complaints (56) or in interactions with police officers (53) T form usage can be deemed highly inappropriate to the point of provocation (57). Since the T form is more commonly used in German and the V form more commonly in French, we hypothesize that:

**H1a:** French speakers will exhibit a preference for a CA using the V form (i.e., “vous”)
**H1b:** German speakers will exhibit a preference for a CA using the T form (i.e., “du”).

In line with users' stated (subjective) preferences, we further posit that T/V distinction will be found to (objectively) cause improved user evaluations (i.e., the CA will be rated more humanlike, or individuals will rate higher likelihood to recommend the CA) when utilizing the T/V distinction appropriate to the given linguaculture (German, French). Accordingly, we hypothesize that:

**H2a:** For French speakers, the use of the formal T/V distinction (i.e., V form) “vous” by CAs will improve individuals' user evaluation scores.
**H2b:** For German speakers, the use of the informal T/V distinction (i.e., T form) “du” by CAs will improve individuals' user evaluation scores.

While a linguaculture exhibits stable traits that are distinct from other linguacultures (44), within-linguaculture differences related to users' age and gender also exist. Age has often been linked to T/V distinction appropriateness, as youth are typically more exposed to emergent cultural trends influencing linguistic evolution (44, 56). For example, older French individuals prefer V form (47), younger German speakers are more likely to use T form (52), and older German speakers may even view T form usage without permission as provocative (56). Gender differences in T/V usage have also been exhibited with French-speaking men more likely to give T form (58) and receive V form than women in interactions with other interlocutors (47), which may correspond to wider differences in language use exhibited between men and women more generally (59) and shifting of gender roles through time (60).

Navigating evolutions in T/V distinction usage in French- and German-speaking linguacultures has often proved difficult for firms and organizations, with changes in T/V distinction used in the workplace and commercial settings (for example, imposition of T form) meeting with scrutiny or resistance (54–56). Additionally, as CAs operate in a channel that typically involves informal, bi-directional communication (i.e., instant text messaging) (61, 62), it is unclear to what extent general cultural norms for formality from strangers in professional settings (41) transfer to the digital environment, and whether variations can be evidenced based on demographic profiling. Thus, while T form has become more widely accepted (63), there remains a body of evidence demonstrating T/V distinction preferences are complex and multifaceted. Therefore, we also examine whether:

**H3:** User Age and Gender jointly moderate the relationship between T/V distinction and Language and user evaluation scores.

## Materials and Methods

### Study Design

To investigate our hypotheses, we conducted a web-based experiment that examined users' preferences for either the T or V form (H1a and H1b), the effects of a healthcare CA's T/V distinction use in two language contexts (H2a and H2b), and the moderating role of participants' demographic profile (i.e., Age and Gender) on user evaluations across the four outcome themes (H3). Participants were randomly allocated to one of two T/V Distinction experimental conditions in their native language; either to the “T condition” (French: “*Tu”*; German: “*Du”*) or to the “V condition” (French: “*Vous”*; German: “*Sie”*). Taken together, the experiment corresponded to a 2 (T form vs. V form) x 2 (French vs. German) full-factorial between-groups design. Following the “Checklist for Reporting of Results of Internet E-Surveys” (64), we outline the study design and procedure in detail.

### Procedure and Participants

In total, 284 participants were recruited from the French (*n* = 136) and German (*n* = 148) speaking parts of Switzerland in September 2019. Individuals ranged in Age from 18 to 84 years old (*M* = 41.9 years, *SD* = 16.6) and 51% were women. Most participants' highest education attained was a high school diploma (64%), 31% had a university degree. Further details on participant background are available in the Supplementary Materials.

Participants were recruited *via* Talk Online Panel GmbH, a European specialist research recruitment company. To compensate for their efforts, participants were rewarded based on a points-based incentive system in line with ESOMAR standards. Participants were sent a survey link *via* e-mail by Talk Online, filtered for their native language, and assigned to a translated (German or French) version of the survey accordingly. After answering further screening (>18 years old, native speakers of either German or French) and demographic (i.e., Age, Gender, and Education) questions, participants were randomly allocated to one of the two T/V Distinction experimental conditions in their native language. After interacting with the respective allocated CA prototype, participants were then redirected to complete the rest of the survey with all outcome variables (user evaluation variables and T/V preference) and debriefed as to the experiment's purpose. In total, the time spent completing the experiment and survey combined ranged from 2.36 mins to 15.15 mins (*M* = 5.65, *SD* = 1.88).

All participants were aware that they could leave the experiment at any time without penalty. Full ethical clearance was given by ETH Zurich Ethics Commission (Ethic's proposal number: 2019-N-127).

### Development of Experimental Stimuli

**Figure 1:**
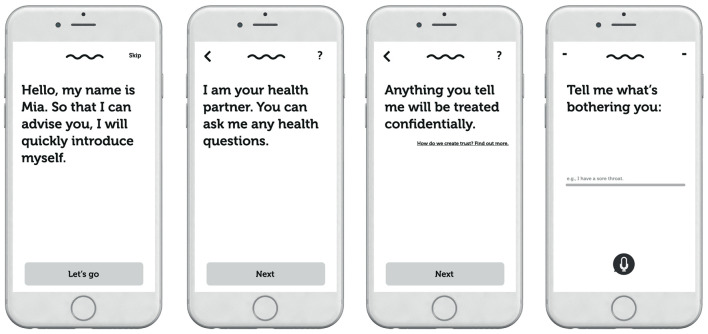
Experimental Stimuli—English Version. Note: “You” form cannot be manipulated in modern English, thus we display one version only.

**Figure 2:**
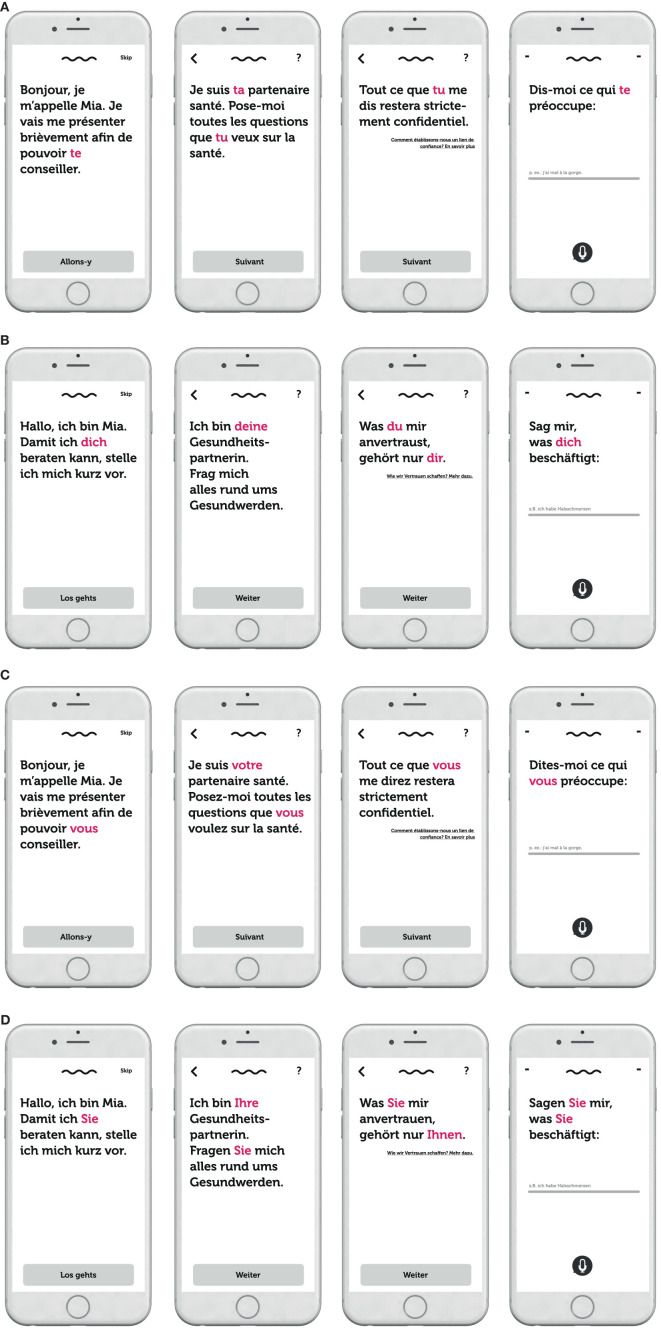
**(A)** Experimental stimuli—French T form. **(B)** Experimental stimuli—German T form. **(C)** Experimental stimuli—French V form. **(D)** Experimental stimuli—German V form. Note: Manipulations highlighted in pink.

The experimental stimuli used were based on a prototype of a healthcare CA developed by a major Swiss health insurance company, created to answer customer queries for health information *via* both text and voice inputs and outputs. Participants could click through the first few conversational turns with the prototype of the CA (named Mia) that was built into a webpage. These conversational turns encompassed the onboarding of the user to the CA interaction rather than the entire medical service. Only the second-person pronouns used by the CA to address the user were manipulated (i.e., the T/V distinction; T form or V form). For purposes of experiment standardization, the interaction with the CA was purely text-based (i.e., no voice in- or output) and followed a rule-based conversational script with predefined answer options (i.e., graphical buttons). An English translation of the app is depicted in Figure 1 and the experimental stimuli for each treatment condition are depicted in Figures 2A–D. The introductory statement to participants (in English) can be found in the Supplementary Material. Manipulation checks confirmed that the experimental conditions functioned as intended.

### Measurement of Outcome Variables

All measurements that focused on measuring attitudes of and perceptions toward the chatbot (to investigate H2a, H2b, and H3) were adapted from established multi-item scales whenever possible (e.g., Social Presence, Trust, etc.; see Table 1). Variables from Davis' (72) Technology Adoption Model were measured based on single-items to reduce the workload for participants as several previous CA studies have used single items for these variables as well (e.g., Liao et al. (73), Oh et al. (74), and Shamekhi et al. (75)). All aforementioned items were measured on 7-point Likert scales ranging from 1 = “Completely disagree” to 7 = “Completely agree.”

**Table 1:** List of measures.

**Construct/Item (Origin)**	**Reference**
**Social Presence** *(7-point Likert, 1 = fully disagree − 7 = fully agree)*	Adapted from Gefen and Straub (65)
I felt a sense of human contact in Mia.	
I felt a sense of personalness in Mia.	
I felt a sense of human warmth in Mia.	
I felt a sense of sociability in Mia.	
I felt a sense of human sensitivity in Mia.	
**Conversational Enjoyment** *(7-point Likert, 1 = fully disagree − 7 = fully agree)*	Adapted from Wixom and Todd, (66)
I enjoyed chatting with Mia	
**Conversational Agent Trust** *(7-point Likert, 1 = fully disagree − 7 = fully agree)*	Adapted from Grohmann, 2009 (67)
I would rely on Mia.	
I would trust Mia.	
Mia is honest.	
**Co-Production** *(7-point Likert, 1 = fully disagree − 7 = fully agree)*	Adapted from Mende and van Doorn (68), Büttgen, Schumann and Ates, (69)
I would openly discuss my health situation with Mia to help her find the best solution for me.	
I would fully cooperate with Mia.	
I would be willing to do my best to achieve a good outcome with Mia.	
**Privacy Concern** *(7-point Likert, 1 = fully disagree − 7 = fully agree)*	Adapted from Kehr, Kowatsch and Wentzel (70)
Compared with others, I am more sensitive about the way digital services handle my personal information.	
To me, it is the most important thing to keep my privacy intact.	
In general, I am very concerned about threats to my personal privacy	
For me, protecting my privacy is a top priority.	
**Perceived Privacy Protection** *(7-point Likert, 1 = fully disagree − 7 = fully agree)*	Adapted from Kehr, Kowatsch and Wentzel (70)
I feel that my privacy would be maintained when interacting with Mia.	
I think that my privacy is preserved when interacting with Mia.	
I would be comfortable with the level of privacy I would have when interacting with Mia.	
**Technology adoption model constructs** *(7-point Likert, 1 = fully disagree − 7 = fully agree)*	Adapted from Wixom and Todd (66)
**Presumed ease of use:** Overall, the service seems easy to use.	
**Presumed usefulness:** I believe that Mia would provide a health service that is useful to me.	
**Presumed service satisfaction:** All things considered, I would be very satisfied with Mia.	
**Net promoter score** *(10-point Likert, 0 = very unlikely − 9 = very likely)*	Adapted from Reichheld (71)
How likely is it that you would recommend Mia to a friend or colleague?	
**Intention Usage** “*Assuming Mia was accessible and fully usable for free, …*	Adapted from Wixom and Todd (66)
**Intention to Use:** “… how likely would you be to use the service?”	
*(11-point Likert, 0 = very unlikely − 10 = very likely)*	
**Intended Usage Frequency:** “… how often would you like to use Mia?”	Own scale
(*1 = Never, 2 = Very rarely (= less often than 1x per month), 3 = Rarely (= at least 1x per month), 4 = Sometimes (= at least 1x per week), 5 = Often (= at least 1x daily), 6 = Very often (= several times daily)*	
**T/V Preference**	Own scale
If you had the choice: Would you prefer Mia to use T- or V-form? *(German: Wenn Sie die Wahl hätten: Wäre es Ihnen lieber, wenn Mia Sie duzen“ oder siezen“ würde?)*	
*I'd prefer it if Mia used … 1 = T-form, 7 = V-for*m	
**Socio-/demographics**	
What is your **gender**? (female, male, other); How **old** are you? (free, text entry); Please indicate your **native language**. (German, French, Other); What is the highest **educational** degree you have? (No school-leaving qualification, Elementary school, Secondary school, High school (Matura), Apprenticeship, University, Bachelor), University (Master), Doctorate, Other)	

*All instructions and items were translated to French and German respectively*.

The Net Promoter Score, which consists of one item by design (71), was measured on a 10-point Likert scale ranging from 0 = “Very unlikely” to 9 = “Very likely.” Intended Usage of the CA [adapted from Wixom and Todd (66)] was measured on an 11-point Likert scale ranging from 0 = “Very unlikely” to 10 = “Very likely.” Intended Usage Frequency (own scale) was measured on a 7-point Likert scale ranging from 1 = “Never” to 7 = “Very often (several times a day).”

Addressing H1a and H1b, we also collected a self-created measure (named T/V Preference) of users' rated subjective preference for either the T or V form (“If you had the choice: Would you rather like Mia to use [T form] or [V form] with you?”) measured on an 11-point Likert scale ranging from 1 = “[T form]” to 11 = “[V form].”

### Statistical Analysis

Prior to analysis, data were filtered for missing responses, language, and other attention and quality checks, with 284 responses included in the final analyses. Where constructs consisted of multiple items, reliability analyses were carried out to discern Cronbach's alpha with all constructs scoring >0.70 threshold (76). Histograms and Q-Q plots were used to test for Gaussian distributions of the dependent variables so that parametric tests could be utilized. To not reduce data points, user Age was included as a continuous variable rather than dichotomized into a categorical variable (77). As T/V preference represents a theoretically distinct concept and does not belong to the same system of variables as user evaluations, separate models were specified for T/V preference and user evaluation outcomes (78). Data were analyzed using R version 4.0.5

#### T/V Preference

In the first model, which investigated users' subjectively stated T/V preference (H1a and H1b), we specified a single ANCOVA model with type III sum of squares with partial eta squared ($\eta_{p}^{2}$) indicating the size of the effect. T/V Distinction, user Language, Gender, and Age were included as independent variables, and all main effects and two-, three- and four-way interactions were investigated (79, 80).

#### User Evaluations

In the second model, we investigated the objective effect of the experimental conditions on all outcome variables (H2a, H2b, and H3). To confirm the suitability of outcome variables from the four themes for MANCOVA analysis, a Pearson's correlation table (Table 2) was first calculated to confirm all outcome variables were below a correlation threshold of *r* = 0.90, indicating variables were sufficiently correlated for multivariate analysis but did not exhibit perfect multicollinearity (81). Following this, we specified a MANCOVA model with type III sum of squares, with partial eta squared ($\eta_{p}^{2}$) and Wilk's Lambda (Λ) indicating effect size. For the dependent variables, we used the user evaluation variables from all outcome themes, and again specified T/V Distinction, user Language, Gender, and Age as independent variables and investigated main effects and two-, three- and four-way interactions (79, 80).

**Table 2:** Pearson correlations of dependent variables.

	**M**	**SD**	**1**	**2**	**3**	**4**	**5**	**6**	**7**	**8**	**9**	**10**	**11**
1. CA trust	4.02	1.69	(0.93)										
2. Co-production	4.34	1.74	0.887^***^	(0.95)									
3. Privacy concern	5.23	1.36	0.120	0.106	(0.88)								
4. Privacy protection	3.95	1.67	0.778^***^	0.738^***^	0.008	(0.96)							
5. Social presence	3.88	1.59	0.720^***^	0.684^***^	0.142^*^	0.686^***^	(0.87)						
6. Convers. enjoyment	4.26	1.68	0.743^***^	0.757^***^	0.052	0.675^***^	0.712^***^	(–)					
7. Perceived ease of use	5.74	1.29	0.483^***^	0.513^***^	0.095	0.403^***^	0.428^***^	0.475^***^	(–)				
8. Perceived usefulness	4.51	1.73	0.788^***^	0.823^***^	0.110	0.693^***^	0.689^***^	0.816^***^	0.524^***^	(–)			
9. Service satisfaction	4.65	1.53	0.718^***^	0.765^***^	0.054	0.637^***^	0.632^***^	0.781^***^	0.531^***^	0.798^***^	(–)		
10. Intended usage	3.15	1.27	0.704^***^	0.689^***^	0.176^**^	0.581^***^	0.571^***^	0.577^***^	0.252^***^	0.610^***^	0.590^***^	(0.70)	
11. Net promoter score	4.78	2.54	0.842^***^	0.856^***^	0.082	0.721^***^	0.751^***^	0.821^***^	0.462^***^	0.831^***^	0.791^***^	0.660^***^	(–)

* *p < 0.05*,

** * p < 0.01*,

*** *p < 0.001 (boldface). In correlation matrix diagonal Cronbach's α are displayed in brackets*.

Where overall significant effects were discerned by the MANCOVA analysis, we followed the procedure outlined in Stevens (82) and Tabachnick and Fidell (81) and utilized identically specified ANCOVA models to confirm whether the significant independent variable(s) found in MANCOVA analysis also held for each independent variable individually (78). Where this was the case, we utilized further Tukey HSD *post-hoc* tests to discern where significant differences between groups existed while controlling for Type I error (81), obtaining slope estimates and confidence intervals. Following guidance outlined by Field, Miles and Field (80), where significant interactions occurred, we investigated the highest-order interactions and not lower-order interactions or main effects (83).

## Results

### ANCOVA Model Results Reveal Significant Main Effect for Language on T/V Preference

The separate ANCOVA model for T/V Preference revealed a significant main effect for Language with a medium effect size (83), *F*_(1, 268)_ = 14.79, *p* = < 0.001, $\eta_{p}^{2}$ = 0.05, with mean scores indicating that French-speaking participants preferred the formal V form (*M* = 7.18, *SE* = 0.32) compared to German-speaking participants who preferred the informal T form (*M* = 4.72, *SE* = 0.30). No other main or interaction effects were discerned. Taken together, both H1a (i.e., that French speakers will exhibit a preference for the formal “vous”) and H1b (i.e., that German speakers will exhibit a preference for the informal “du”) are fully supported.

### MANCOVA Model Results Reveals Significant Four-Way-Interaction Effect on User Evaluations

The MANCOVA model specified using all outcome variables showed no significant main effects, however, interaction effects were found for T/V Distinction and Gender (*Wilks'* λ = 0.924, *F*_(11, 258)_ = 1.939, *p* = 0.035, $\eta_{p}^{2}$ = 0.076), T/V Distinction, Gender and Age (*Wilks'* λ = 0.921, *F*_(11, 258)_ = 2.014, *p* = 0.027, $\eta_{p}^{2}$ = 0.079), T/V Distinction, Language, and Gender (*Wilks'* λ = 0.907, *F*_(11, 258)_ = 2.404, *p* = 0.007, $\eta_{p}^{2}$ = 0.093), and T/V Distinction, Language, Gender, and Age (*Wilks'* λ = 0.903, *F*_(11, 258)_ = 2.531, *p* = 0.005, $\eta_{p}^{2}$ = 0.097) showing effect sizes ranging from medium to small (83). Table 3 yields an overview of the MANCOVA model results. Additionally, as the continuous independent variable Age was found significant, we also investigated if multivariate curvilinear trends were present by including a 2nd degree polynomial term for Age in the above model, however, no significant improvement to model fit was found (see Supplementary Materials), confirming suitability for linear analyses.

**Table 3:** MANCOVA model results.

**MANCOVA**	***df***	***Wilks' λ***	***F***	***p***	*** $\eta_{p}^{2}$ ***
**Main effects**					
T/V distinction	11	0.962	0.917	0.524	0.038
Language	11	0.940	1.500	0.131	0.060
Age	11	0.935	1.622	0.093	0.065
Gender	11	0.972	0.655	0.781	0.021
**Two-way interaction effects**					
T/V distinction * language	11	0.978	0.518	0.891	0.022
T/V distinction * age	11	0.964	0.872	0.569	0.036
T/V distinction * gender	**11**	**0.924**	**1.939**	**0.035**	**0.076**
Language * age	11	0.955	1.102	0.360	0.045
Language * gender	11	0.970	0.732	0.708	0.030
Gender * age	11	0.979	0.503	0.900	0.021
**Three-way interaction effects**					
T/V distinction * language * age	11	0.975	0.601	0.828	0.025
T/V distinction * language * gender	**11**	**0.907**	**2.404**	**0.007**	**0.093**
T/V distinction * gender * age	**11**	**0.921**	**2.014**	**0.027**	**0.079**
Language * gender * age	11	0.980	0.481	0.914	0.020
**Four-way interaction effects**					
T/V Distinction * language * gender * age	**11**	**0.903**	**2.531**	**0.005**	**0.097**

*Significant values at p < 0.05 are in boldface*.

### Follow-Up ANCOVA Models

As the MANCOVA discerned a significant four-way interaction between T/V Distinction, Language, Gender, and Age, ANCOVA models for each outcome variable were specified in the same manner (78, 81, 82). In each of the ANCOVA models across all outcome variables, the significant four-way interaction was again confirmed with the exceptions of Privacy Concern (PC) (*p* = 0.120) and Perceived Ease of Use (PEOU) (*p* = 0.400) which were insignificant and therefore not further analyzed in the *post-hoc* analyses. An excerpt of the ANCOVA models results for the four-way interaction effect can be found in Table 4.

**Table 4:** ANCOVA models results for four-way interaction between T/V Distinction, language, gender, and age per outcome variable.

**Theme**	**Dependent variable**	***F***	***p***	*** $\eta_{p}^{2}$ ***
**Sociability**	Social presence	**8.447**	**0.004**	**0.031**
	Conversational enjoyment	**5.769**	**0.017**	**0.021**
				
**CA-User Collaboration**	Conversational agent trust	**4.667**	**0.032**	**0.017**
	Privacy concern	2.421	0.120	0.009
	Perceived privacy protection	**6.731**	**0.010**	**0.024**
	Co-production	**7.997**	**0.005**	**0.029**
				
**Service Evaluation**	Perceived ease of use	0.710	0.400	0.003
	Perceived usefulness	**12.480**	**0.000**	**0.044**
	Service satisfaction	**4.728**	**0.031**	**0.017**
				
**Behavioral Intentions**	Intended usage	**9.295**	**0.003**	**0.034**
	Net Promoter score	**10.923**	**0.001**	**0.039**

*Significant values at p < 0.05 are in boldface. Only four-way interaction results are displayed*.

### *Post-hoc* Tests Reveal Consistent Four-Way Interaction Pattern for Nine Out of 11 Outcome Variables

**Figure 3:**
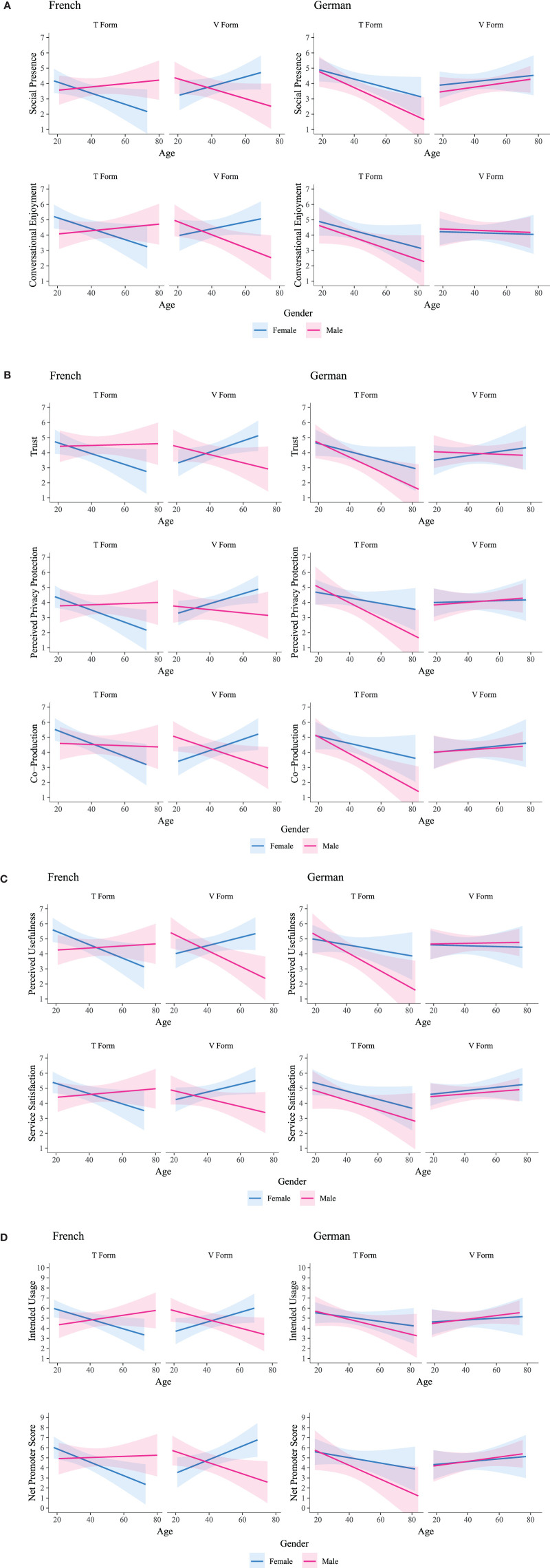
**(A)** Sociability Outcomes. **(B)** CA-User Collaboration Outcomes. **(C)** Service Evaluation Outcomes. **(D)** Behavioral Intention Outcomes. Note: Four-way interaction graphs.

To further explore the four-way interactions confirmed in the ANCOVA models, subsequent *post-hoc* Tukey HSD tests were conducted to estimate the overall slope coefficients and confidence intervals for T/V Distinction, Language, Gender by Age; graphically represented in Figures 3A–D and summarized in Tables 5A–D. The results showed a four-way interaction between T/V Distinction, Gender, Language, and Age for all included user evaluation variables with a consistent pattern across all outcome themes. The pattern evidenced is described in the following passages using the outcomes of Net Promoter Score (NPS) and Social Presence as examples:

**Table 5:** Slope coefficients.

**Measure**	**T/V**	**Language**	**Gender**	**Age Slope**	**SE**	**Lower CI**	**Upper CI**
**(A)**							
**(i) Sociability**							
*Social Presence*	T	FR	F	−0.036	0.018	−0.071	−0.002
	V	FR	F	0.031	0.018	−0.004	0.065
	T	GER	F	−0.028	0.015	−0.057	0.002
	V	GER	F	0.011	0.016	−0.020	0.042
	T	FR	M	0.011	0.015	−0.019	0.041
	V	FR	M	−0.033	0.017	−0.066	0.000
	T	GER	M	−0.047	0.018	−0.083	−0.012
	V	GER	M	0.015	0.014	−0.013	0.042
*Conversational Enjoyment*	T	FR	F	−0.036	0.019	−0.073	0.001
	V	FR	F	0.023	0.019	−0.015	0.060
	T	GER	F	−0.027	0.016	−0.060	0.005
	V	GER	F	−0.003	0.017	−0.037	0.031
	T	FR	M	0.011	0.017	−0.022	0.043
	V	FR	M	−0.043	0.018	−0.078	−0.007
	T	GER	M	−0.036	0.019	−0.074	0.002
	V	GER	M	−0.004	0.015	−0.034	0.025
**(B)**							
**(ii) CA-User Collaboration**							
*Trust in CA*	T	FR	F	−0.036	0.019	−0.073	0.001
	V	FR	F	0.037	0.019	0.000	0.075
	T	GER	F	−0.027	0.016	−0.059	0.005
	V	GER	F	0.014	0.017	−0.020	0.047
	T	FR	M	0.003	0.017	−0.030	0.035
	V	FR	M	−0.027	0.018	−0.063	0.008
	T	GER	M	−0.048	0.019	−0.086	−0.011
	V	GER	M	−0.004	0.015	−0.034	0.025
*Perceived Privacy Protection*	T	FR	F	−0.040	0.019	−0.077	−0.004
	V	FR	F	0.033	0.019	−0.004	0.070
	T	GER	F	−0.018	0.016	−0.050	0.014
	V	GER	F	0.003	0.017	−0.030	0.036
	T	FR	M	0.004	0.017	−0.029	0.036
	V	FR	M	−0.011	0.018	−0.046	0.024
	T	GER	M	−0.053	0.019	−0.090	−0.015
	V	GER	M	0.008	0.015	−0.021	0.037
*Co*-*Production Behavior*	T	FR	F	−0.042	0.019	−0.080	−0.005
	V	FR	F	0.038	0.019	0.000	0.075
	T	GER	F	−0.024	0.017	−0.056	0.009
	V	GER	F	0.010	0.017	−0.024	0.045
	T	FR	M	−0.004	0.017	−0.037	0.029
	V	FR	M	−0.037	0.018	−0.073	−0.001
	T	GER	M	−0.057	0.020	−0.095	−0.018
	V	GER	M	0.007	0.015	−0.023	0.037
**(C)**							
**(iii) Service evaluation**							
*Perceived Usefulness*	T	FR	F	−0.045	0.019	−0.082	−0.007
	V	FR	F	0.028	0.019	−0.010	0.065
	T	GER	F	−0.018	0.017	−0.050	0.015
	V	GER	F	−0.003	0.017	−0.037	0.031
	T	FR	M	0.007	0.017	−0.026	0.040
	V	FR	M	−0.053	0.018	−0.089	−0.018
	T	GER	M	−0.057	0.019	−0.096	−0.019
	V	GER	M	0.002	0.015	−0.028	0.032
*Service Satisfaction*	T	FR	F	−0.034	0.017	−0.068	0.000
	V	FR	F	0.026	0.017	−0.008	0.060
	T	GER	F	−0.027	0.015	−0.056	0.002
	V	GER	F	0.011	0.016	−0.020	0.042
	T	FR	M	0.010	0.015	−0.020	0.039
	V	FR	M	−0.026	0.016	−0.059	0.006
	T	GER	M	−0.032	0.018	−0.066	0.003
	V	GER	M	0.008	0.014	−0.019	0.035
**(D)**
**(iv) Behavioral Intentions**
*Intended Usage*	T	FR	F	−0.025	0.014	−0.053	0.004
	V	FR	F	0.023	0.014	−0.005	0.051
	T	GER	F	−0.001	0.013	−0.026	0.023
	V	GER	F	0.007	0.013	−0.018	0.033
	T	FR	M	0.025	0.013	0.000	0.050
	V	FR	M	−0.017	0.014	−0.044	0.010
	T	GER	M	−0.021	0.015	−0.050	0.007
	V	GER	M	0.013	0.011	−0.009	0.036
*Net Promoter Score*	T	FR	F	−0.067	0.028	−0.122	−0.011
	V	FR	F	0.068	0.028	0.012	0.123
	T	GER	F	−0.027	0.024	−0.075	0.021
	V	GER	F	0.014	0.026	−0.037	0.064
	T	FR	M	0.006	0.025	−0.043	0.054
	V	FR	M	−0.055	0.027	−0.108	−0.002
	T	GER	M	−0.069	0.029	−0.125	−0.012
	V	GER	M	0.021	0.022	−0.023	0.065

For French speakers, when the informal T form (“*Tu”*) was used, higher user evaluation scores were generated for younger women and older men, whereas when the formal V form (“*Vous”*) was used, higher user evaluation scores were generated for younger men and older women respectively. For example, for French women in the T condition, as their Age increased, Net Promoter Score (β = −0.067, *SE* = 0.028) and Social Presence (β = −0.036, *SE* = 0.018) scored lower (i.e., individuals rated lower likelihood to recommend Mia to friends or relatives and Mia felt less humanlike) whereas French women in the V condition scored higher (i.e., individuals rated higher likelihood to recommend Mia to friends or relatives and Mia felt more humanlike) with Net Promoter Score (β = 0.068, *SE* = 0.028) and Social Presence (β = 0.031, *SE* = 0.018). Conversely, for French men in the T condition, as their Age increased, Net Promoter Score (β = 0.006, *SE* = 0.025) and Social Presence (β = 0.011, *SE* = 0.015) rated higher (i.e., individuals rated higher likelihood to recommend Mia to friends or relatives and Mia felt more humanlike), whereas French men in the V condition scored lower (i.e., individuals rated lower likelihood to recommend Mia to friends or relatives and Mia felt less humanlike) with Net Promoter Score (β = −0.055, *SE* = 0.022) and Social Presence (β = −0.033, *SE* = 0.017) respectively.

For German speakers, when the informal T form (“*Du”*) was used, younger users' evaluation scores rated comparably regardless of Gender, however, as individuals' Age increased, the use of “*Du”* resulted in relatively lower user evaluation scores, and this effect was even more pronounced for men. Whereas, in the formal V condition (“*Sie”*), user evaluation scores were relatively stable, regardless of Gender, and showed only a slight influence of Age. For example, in the informal T condition as users' Age increased, Net Promoter Score (β = 0.027, *SE* = 0.024) and Social Presence (β = 0.028, *SE* = 0.015) rated lower for women, and even lower for men (i.e., individuals rated lower likelihood to recommend Mia to friends or relatives and Mia felt less humanlike) with Net Promoter Score (β = 0.069, *SE* = 0.029) and Social Presence (β = 0.047, SE = 0.018) respectively. Whereas in the formal V condition trends were comparably stable between Genders as users' Age increased [e.g., Net Promoter Score: male (β = 0.021, *SE* = 0.022) vs. female (β = 0.014, *SE* = 0.026) scores; Social Presence: male (β = 0.015, *SE* = 0.014) vs. female (β = 0.011, *SE* = 0.016)] scores (i.e., for both genders as individuals' age increased, individuals stated a slight increased likelihood to recommend Mia to friends or relatives and rated Mia more humanlike).

### Pairwise Comparisons

Further Tukey pairwise comparisons were conducted with a summary of the significant differences in the slope trend between groups (as calculated by subtracting comparison group, β_*c*_, from the reference group, β_*r*_) summarized in Table 6. Findings outlined that as individuals' Age increased, French-speaking women in the V condition exhibited significantly higher user evaluation scores than German-speaking men in the T condition for Trust (β_*r*_*-*β_*c*_ = 0.086, *SE* = 0.027, *p* = 0.034), Perceived Privacy Protection (β_*r*_*-*β_*c*_ = 0.086, *SE* = 0.027, *p* = 0.032), Co-Production (β_*r*_*-*β_*c*_ = 0.094, *SE* = 0.027, *p* = 0.015), Social Presence (β_*r*_*-*β_*c*_ = 0.078, *SE* = 0.025, *p* = 0.042), Perceived Usefulness (β_*r*_*-*β_*c*_ = 0.085, *SE* = 0.027, *p* = 0.041) and Net Promoter Score (β_*r*_*-*β_*c*_ = 0.136, *SE* = 0.039, *p* = 0.018). Further significant differences were found in a similar pattern with French-speaking women in the V condition rating Net Promoter Score (β_*r*_*-*β_*c*_ = 0.123, *SE* = 0.039, *p* = 0.038) and Perceived Usefulness (β_*r*_*-*β_*c*_ = 0.081, *SE* = 0.026, *p* = 0.047) higher as their Age increased than French-speaking men in the V condition. For Net Promoter Score (β_*r*_*-*β_*c*_ = 0.134, *SE* = 0.040, *p* = 0.019), French-speaking women in the V condition also significantly differed from French-speaking women in the T condition as their Age increased. Other marginally significant (*p* < 0.1) pairwise comparisons were also evidenced for Co-Production.

**Table 6:** Tukey HSD pairwise comparisons.

**DV**	**Reference group**	**Comparison group(s)**	***β_r_* - β_*c*_**	***SE***	***p***
Conversational Agent Trust	V, FR, F	T, GER, M	**0.086**	**0.027**	**0.034**
Perceived Privacy Protection	V, FR, F	T, GER, M	**0.086**	**0.027**	**0.032**
Co-Production	V, FR, F	T, GER, M	**0.094**	**0.027**	**0.015**
	V, FR, F	V, FR, M	0.075	0.027	0.097
	V, FR, F	T, FR, F	0.080	0.027	0.067
Social Presence	V, FR, F	T, GER, M	**0.078**	**0.025**	**0.042**
Perceived Usefulness	V, FR, F	T, GER, M	**0.085**	**0.027**	**0.041**
	V, FR, F	V, FR, M	**0.081**	**0.026**	**0.047**
Net Promoter Score	V, FR, F	T, GER, M	**0.136**	**0.039**	**0.018**
	V, FR, F	V, FR, M	**0.123**	**0.039**	**0.038**
	V, FR, F	T, FR, F	**0.134**	**0.040**	**0.019**

*Significant values at p < 0.05 are in boldface*.

Taken together, we find partial support for H2a (i.e., that CA use of formal “vous” with French-speaking participants causes higher user evaluation scores) as this occurred only for certain user groups (younger men, older women). Similarly, we find partial support for H2b (i.e., that CA use of informal “du” with German-speaking participants causes higher user evaluation scores) as this again occurred only for certain user groups (younger men, younger women). Additionally, we can fully confirm H3 (i.e., that user Age and Gender jointly moderate the relationship between T/V distinction and Language and user evaluation scores) as in each language setting there was not a single T/V form that caused highest user evaluation outcomes, rather, highest user evaluation scores depend on both the Language used and the users' demographic profile (i.e., Age, Gender). The results, therefore, confirm the importance of both linguaculture *per se* (with differences evidenced between both French and German speakers) as well as the importance of demographic profiling of users within a linguaculture (with differences evidenced by Gender and Age).

## Discussion

### Theoretical Contributions

In this study, across all four user evaluation outcome themes, we have shown that the term of address (T/V distinction) employed by CAs varies in suitability both between linguacultures (i.e., French, German) but also within linguacultures by demographic profiling (i.e., Gender, Age). To the authors' knowledge, this is the first time T/V distinction has been linked to a wide range of CA-relevant outcomes, making three main theoretical contributions relevant to the design of CA-based digital health interventions.

First, regarding the outcome themes [i.e., (i) Sociability, (ii) CA-User Collaboration, (iii) Service Evaluation, and (iv) Behavioral Intentions], we demonstrate how the term of address has a wide-reaching impact on a variety of user evaluation outcomes. Findings from the (i) Sociability theme underscore the importance of anthropomorphism (18, 84) and our research begins the process of linking specific linguistic cues to perceptions of humanness. As considerable angst surrounds T/V distinction usage (52), it is likely that when presented with the T/V form that most closely matches socially-based expectations, Mia (the CA) was perceived as a more efficacious and socially experienced dyadic partner and thus higher ratings of Social Presence and Conversational Enjoyment were found. For the Service Evaluation theme, this may explain why Perceived Usefulness and Service Satisfaction were significant while Perceived Ease of Use was not: Individuals likely evaluated the former two variables in terms of their relationship with Mia (the CA) as a dyadic partner, whereas the latter was based on the app interface. Additionally, as enabling working relationships between users and healthcare CAs has been a widely desired outcome (15), findings for the (ii) CA-User Collaboration theme show how appropriate use of T/V form facilitates CA-user collaboration by increasing Trust, Perceived Privacy Protection, and Co-Production (i.e., ability to work well with the CA). It may be that for those groups where T form caused higher user evaluation scores, its use created a greater sense of CA-User relatedness (48) facilitating feelings of trust and security, whereas, for those groups where V form caused higher user evaluation scores, its usage conveys themes of power asymmetry (48) and thus connotations of formality, professionalism, or respect.

Second, our findings highlight how evaluative processes vary in a distinct manner between user groups. Pairwise comparisons revealed that for older French-speaking women the use of V form was something relatively value-adding (causing improvements in user evaluation scores), whereas for older German-speaking men the lack of V form use (i.e., using T form) was value-reducing (causing reductions in user evaluation scores). As memory is organized in associative networks (85), it may be that semantic cues in T or V forms convey different levels of interpersonal closeness, formality, or professionalism (48), which serve to confirm or disconfirm expectations for different user groups (51). Previous research has shown, that age, social status, relationships (friends, colleagues, acquaintances) (50, 53), and physical location of conversation (52) are all part of an evaluative process influencing the selection of a cultural script (44), and in turn, reception of linguistic devices used (45) such as terms of address (41). We posit therefore that CA use of T/V distinction confirms the appropriate cultural script for users (based on some underlying implicit need) and facilitates improved user evaluations.

Third, regarding users' subjectively stated T/V preference, we reinforce prior findings showing that preferences vary between linguacultures (41, 49), as French speakers rated a significantly higher preference for the V form. This is likely due to the V form being relatively more common in French in a variety of usage contexts in everyday life, whereas in German it is used less frequently and typically in professional settings (49). Surprisingly, Age was not significantly linked to T/V Preference, in contrast to previous findings (50), which could be taken as evidence of the globalization effect on T/V distinction (43). We would however posit an alternative reasoning: Results from our experiment showed contradictions between users' subjectively stated preferences and objective experimental effects generating best user evaluations. For example, despite French-speaking users stating a preference for V form and Age having no significant influence on T/V preference, results showed that, for older French-speaking men, highest user evaluation scores were generated by T form. Thus, despite T/V Preference not significantly differing by users' Age and Gender, we can see that T/V Distinction usage nonetheless has important effects on user evaluation outcomes, moderated by participants' Age and Gender, in the two language settings. This contradiction in stated preferences in healthcare is known as “hypothetical bias,” whereby individuals' reported preferences are not congruent to outcomes in a real situation (86, 87) thus underlining the importance of using experimental research when designing healthcare CAs.

### Managerial Implications

The present research offers the following managerially relevant contributions:

First, we demonstrate that unique linguistic-cultural features are still of relevance in the era of globalization. This is of importance as an assumption in some practice-led circles has been that greater globalization will lead to the homogenization of preferences between nations (43), and in healthcare, this is often reflected in standardized approaches when transferring knowledge across borders (88). Indeed, in pursuit of scaling-up health interventions, rapid implementation is often encouraged to address the digital divide between developed and developing nations (89), whilst at the same time, greater personalization of healthcare is known to be both more beneficial as well as a strength readily delivered by digital tools such as CAs (13). While not wishing to dissuade the important rollout of new technologies, our research suggests that considering unique linguistic-cultural features within a linguaculture (such as T/V distinction) and optimizing CA dialogues accordingly would be a worthwhile step: Particularly as relevant demographic user characteristics can be readily elicited early in dialogues with the CA and subsequently utilized for personalization (90).

Second, our findings show that the term of address used significantly and robustly affects managerially relevant outcomes from the Behavioral Intentions theme. Pairwise comparisons revealed a significant effect of CA use of T/V distinction on the Net Promoter Score, a construct strongly linked to commercial success (91), that is also becoming more widely used in healthcare (12, 92). Additionally, pairwise comparisons revealed a significant effect of address form on participants' Intention to Use the CA in the future. Although intentions may not (always) lead to behavior (93), forging positive attitudes, associations, and intentions are still important steps in behavior change (94), helping to create engaging user experiences that patients adhere to, which is, ultimately, vital for treatment success (95). Our results, therefore, demonstrate a practical method by which practitioners can improve peer-network recommendations and adoption of their CA-based digital health interventions.

In summary, while maintaining long-term CA-user relationships will likely vary on many factors, T/V distinction and other linguistic devices may have a greater role in first impression management, and potentially also longer-term interactions too, than previously realized.

### Limitations and Future Research

Although greatly elucidating how terms of address used by CAs affect user evaluations, further research can build upon our findings in several research directions (RDs). The current research examined two unique linguacultures of French and German speakers, however, within a single multi-lingual nation of Switzerland. Switzerland's unique context allowed us to mitigate potential bias from other inter-national/cultural differences, for example, between different nation-states (96, 97). However, users from Switzerland may be more culturally homogenous than say French speakers from France and German speakers from Germany, which may have caused less variation in user evaluations. Additionally, while no dialect was employed in the app (i.e., standard French and German was used), it could be that individuals from non-geographically proximal locations to Switzerland that historically have used different dialects (for example, French-speakers in Belgium or German-speakers in northern Germany) may exhibit some variation in evaluations. Further research should therefore examine users from two distinct nations, which may confirm trends identified (RD1).

Another consideration would be a comparison of effect sizes. The current research discerned a medium effect size of Language on (subjectively stated) T/V Preference and a small effect size of (objectively manipulated/controlled) T/V distinction, Language, Gender, and Age on user evaluations. Although previous CA-based research has highlighted that use of users' first names (as the term of address) causes significantly improved CA evaluations, no effect size was reported by the authors (25). Additionally, papers on terms of address in linguistics fields are typically qualitative, using methodologies such as Natural Semantic Metalanguage (49) or ethnographic observation (52). Thus, comparisons of our results with published research are difficult. Some studies including effect sizes of language-based cues used by CAs are available in broader research outside terms of address, for example, Schuetzler, Grimes, and Giboney (1) examined tailored vs. generic communication styles and found tailored communication significantly improved perceived humanness, with a large effect size. Additionally, Rietz et al. (98) found CA use of social cues in dialogues (use of emojis, short pauses) significantly improved ratings of usability outcomes, with small effect sizes. As CA-research is in its infancy, we would therefore encourage future researchers to publish details on experimental manipulations used and effect sizes discerned. In such a way, a taxonomy of linguistic features can be built (RD2) acting as a research reference and practical guide for CA designers. A next step in building this taxonomy, following the current paper, could be to examine T/V distinction (or related facets of speech etiquette) in non-Latin languages (RD3). If findings from the current study were also confirmed in other linguacultures, global universals in CA design could be established, which may enable faster adaptation of health interventions internationally. This would fit with recent calls from the World Health Organization to scale-up CAs (14), and provide an interesting research opportunity to investigate CA speech etiquette in linguacultures around the globe.

An additional consideration regards temporal aspects of the healthcare CA used in the current experiment. Individuals using Mia were interacting for the first time, and a variety of more complex rituals govern first impression management (33). Research from CA literature has shown that long-term relationships typically have more social-emotional components to them (33), which is particularly the case during long-term CA-led interventions to address chronic diseases (22). Therefore, whilst we can be confident how to make a positive first impression based on the current results, which are particularly applicable to health services such as symptom checking (3), CA T/V distinction appropriateness may change over time for longitudinal interventions, mirroring aspects of relationship development in non-digital contexts (52). Further research may therefore wish to investigate longitudinal aspects of CA term of address use (RD4). In a similar vein, while our results confirmed the importance of Age as a moderator, our results cannot definitively state whether this is due to generational differences that will dissipate over time (50), or whether as individuals age they are socialized into utilizing certain terms of address (63). Moving forward, it will therefore be fascinating to examine how humans and machines address each other, with the advent of newer technologies and longitudinal data (RD5).

Lastly, for certain variables (PC, PEOU), trends were identified that appear to similarly fit the pattern identified for other outcome variables, however, were not statistically significant. This may be because the current user experience with Mia (the CA) was too brief, not realistic enough, or the current sample size was not large enough to detect effects. Future research may wish to examine these variables again with a fully operational prototype (RD6) or larger sample size (RD7).

## Conclusion

Conversational agents are driven by the ability to communicate effectively with users, in ways that connect with them and build working alliances. In a variety of linguacultures globally, T/V distinction represents an ever-salient term of address and an important linguistic device. By conforming to users' expectations regarding facets of speech-etiquette and language-use more generally, designers of CAs can shape engaging user experiences. For healthcare CAs this is particularly vital, as it facilitates greater adherence to the treatment plans they help deliver. The current paper, therefore, contributes to a greater understanding of CA linguistics from an international perspective, as well as providing practical steps to design and adapt digital health interventions cross-culturally: Ultimately aspiring to begin addressing the digital divide between developing and developed nations by increasing the effectiveness of CA use globally.

## Data Availability Statement

The raw data supporting the conclusions of this article will be made available by the authors, without undue reservation.

## Ethics Statement

The studies involving human participants were reviewed and approved by ETH Zurich (Ethic's proposal number: 2019-N-127). The patients/participants provided their written informed consent to participate in this study.

## Author Contributions

JO and MN were equally responsible for conceptualization, methodology, data curation, and drafting the manuscript. JO was responsible for data analysis. MN for project administration, and funding acquisition. All authors reviewed and approved the manuscript before submission.

## Funding

Funding for recruiting participants within the MIA project was provided by CSS insurance.

## Conflict of Interest

JO, MN, and FvW are affiliated with the Center for Digital Health Interventions (www.c4dhi.org), a joint initiative of the Department of Management, Technology, and Economics at ETH Zurich and the Institute of Technology Management at the University of St. Gallen, which is funded in part by the Swiss health insurance CSS Versicherungen. CSS provided funding for recruiting participants but was neither involved in any aspect of the study design, data analysis nor manuscript preparation.

## Publisher's Note

All claims expressed in this article are solely those of the authors and do not necessarily represent those of their affiliated organizations, or those of the publisher, the editors and the reviewers. Any product that may be evaluated in this article, or claim that may be made by its manufacturer, is not guaranteed or endorsed by the publisher.

## Footnotes

^1^ https://babylonhealth.com

^2^ https://ada.com/de/

^3^ https://bot.hiv.gov/

^4^ https://woebothealth.com/

^5^ https://wysa.io/

^6^ https://www.x2ai.com/

^7^https://florence.chat/; Cottrell et al. (11).

^8^https://www.lark.com/; Stein and Brooks (12).
