# Supplementary material for: The Terms of “You(s)”: How the Term of Address Used by Conversational Agents Influences User Evaluations in French and German Linguaculture
Source: Front Public Health. 2022 Jan 5;9:691595. doi: 10.3389/fpubh.2021.691595 (PMC8767023; doi:10.3389/fpubh.2021.691595)
Supplement: Supplementary file 1 [file Data_Sheet_1.PDF]

Supplementary Material

For the interested reader, further details regarding user evaluation outcome themes, the introductory statement and testing for curvilinear relationships in the supplementary material:

1 Outcome Themes and Variable Selection

For the first theme, Sociability, the variables Social Presence and Conversational Enjoyment were selected. Originally, Social Presence was a concept used to assess users‘ sense of interacting with another human being in computer-mediated interactions between humans (1) and found to predict, for example, trust (2), satisfaction (3,4) and purchasing intentions in service encounters (5). Conceptualizing CAs as anthropomorphic entities under the "Computers Are Social Actors" paradigm (6), Social Presence and Conversational Enjoyment have become commonly discussed elements in research related to CAs (7) and increased CA adoption (8). However, while previous work found that a sense of Social Presence can be induced by manipulating a CA to use more social-oriented (4), more polite (3) or more informal (7) language, effects of the T/V form on users' sense of Social Presence or Conversational Enjoyment have not been studied before.

The second theme, CA-User Collaboration, comprises the variables Trust, Perceived Privacy Protection, Privacy Concern and Co-Production. Trust has been acknowledged a key factor in successful patient-doctor communication (9) and a prerequisite for intimacy and the facilitation of self-disclosure (10). Similarly, previous human-chatbot studies have found how verbal anthropomorphic cues facilitate Trust leading to increased self-disclosure (11). However, self-disclosure only occurs when individual perceptions of the privacy and safety of their information is guaranteed, as stated in Communication Privacy Management theory (12). Therefore, Privacy Concerns and Perceived Privacy Protection are of high importance alongside Trust (13) in facilitating CA-User Interaction and cooperation with the CA (14,15). The final measure in this group therefore is Co-Production, a measure widely used in services research, reflecting the degree to which an individual feels empowered to co-create their service experience alongside other service personnel (16). As patient empowerment is vital in healthcare settings as a facilitator of patient independence, self-management and self-efficacy (17), it is therefore likewise important to understand whether users experiences of co-producing with CAs differ.

The third theme, Service Evaluation, entails three variables: users' Perceived Ease of Use (PEOU), Perceived Usefulness (PU) of the service and Service Satisfaction. Together with Conversational Enjoyment (cf. Sociability theme) they represent the complete Technology Adoption Model first introduced by Davis in 1989 (18) which aims to provide a measurable understanding of how behavioral intentions of technology adoption (i.e., Intended Usage, cf. Behavioral Intentions theme) are influenced by attitude constructs. However, whereas perceived Conversational Enjoyment relates to a hedonic value of using the CA as reflected in the Sociability theme, PEOU, PU and Service Satisfaction encompass utilitarian features of CA usage and are therefore encapsulated in the (iii) Service Evaluation theme. Both facets (utilitarian and hedonic) have been found to have significant effects on CA effectiveness (19–21). Yet, while previous studies have demonstrated that the merely interacting with a CA, when compared to control groups that did not involve such interactions, significantly improved users' attitudes towards the service provided by the CA (22) and towards the

CA itself (23), there is scarcity of research on how different chatbot designs such as its use of T/V distinction affect any of these concepts.

The fourth and last theme, Behavioral Intentions, consists of two variables: Intended Usage (IU) and the Net Promoter Score (NPS). Measuring IU as a proxy for actual usage has been widely accepted in technology adoption studies since the relationship between IU and actual usage is well established empirically (24). NPS is a popular metric in the business world due to its simplicity and ability to capture loyalty related facets of intention (25), which may be particularly important in CA-User Interaction that contain both emotive and cognitive components (14), as also reflected in its increasing usage to evaluate coaching-based digital health interventions (26,27). While a plethora of CA studies have already linked specific design cues to users' behavioral intentions, including verbal cues (28), linguistic cues such as the use of formal vs. informal language (7), anthropomorphic visual cues (28), and paralinguistic cues (8), no study so far has investigated the role of T/V distinction.

2 Introductory Statement

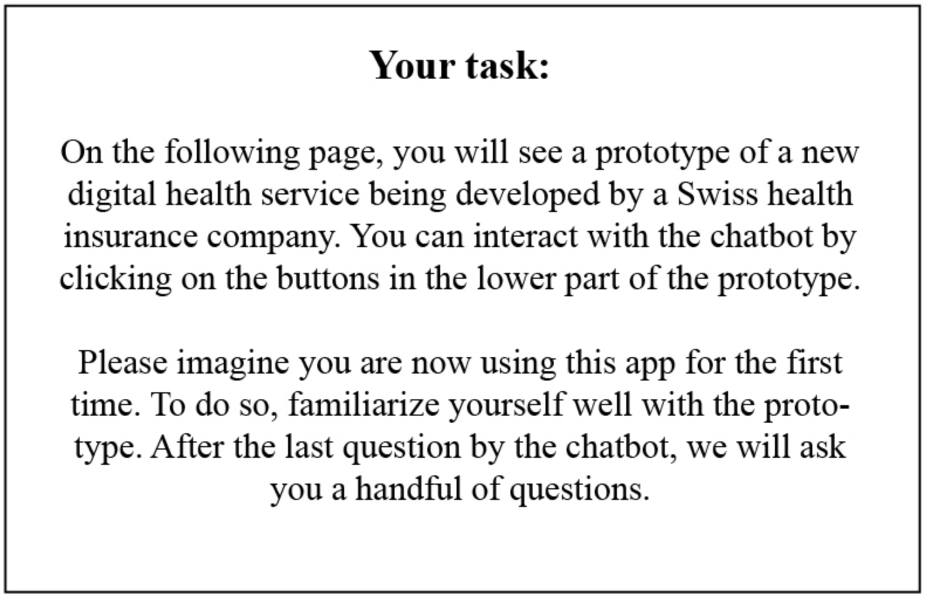

Your task:

On the following page, you will see a prototype of a new digital health service being developed by a Swiss health insurance company. You can interact with the chatbot by clicking on the buttons in the lower part of the prototype.

Please imagine you are now using this app for the first time. To do so, familiarize yourself well with the proto-type. After the last question by the chatbot, we will ask you a handful of questions.

Figure 1 | Participant introductory statement (translated)

3 Exploring Curvilinear Relationships

To test for the existence of curvilinear relationships, we compared two MANCOVA models with type III sum of squares. The first model was identical to that of section 2.5.2 outlined in the main paper, user evaluations were specified as the dependent variable, and T/V Distinction, user Language, Gender and Age were specified as independent variables. All main and interaction terms were included. The second model was specified similarly, except we included an additional 2nd degree polynomial term for the continuous variable Age which could confirm if any quadratic trends were evidenced in the data. The two models were compared subsequently with ANOVA, which found Model 2 did not fit significantly better than Model 1 ($F(176, 2772) = 1.111$, $p = .158$), with generalized variance explained $|S| = 1.004$ and $|S| = 1.012$ respectively (29). Thus, we confirm

suitability for linear analyses. A graphical comparison of the models can be viewed in Figures 2 (A-B). For graphing purposes a composite variable was created based on all user evaluation scores used in the MANCOVA analyses):

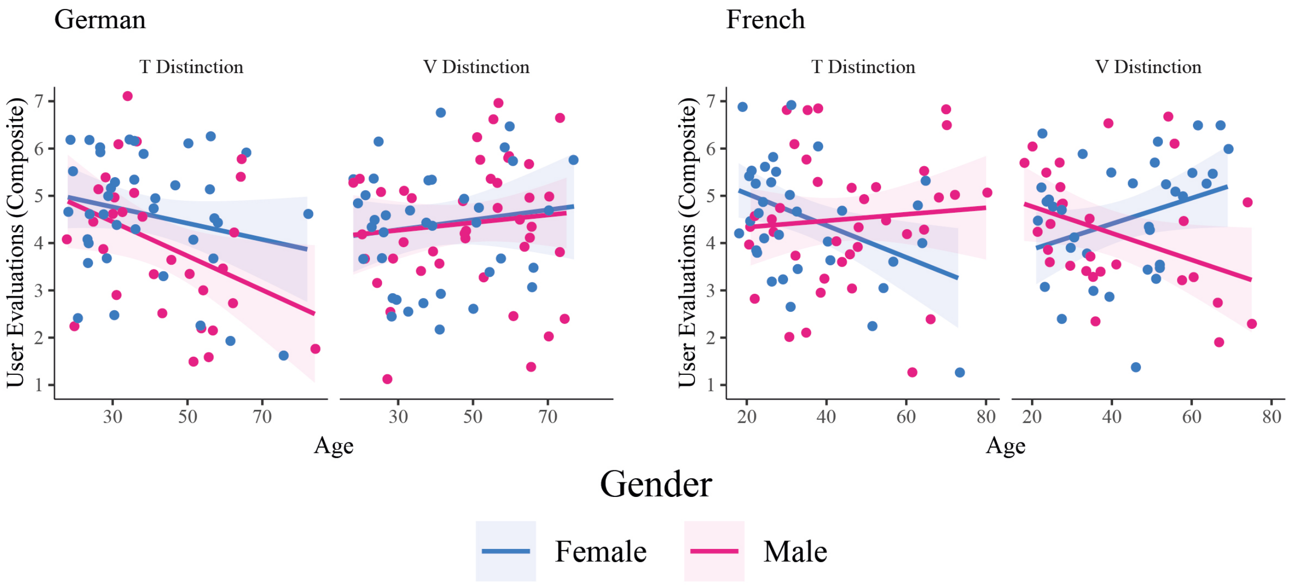

Figure 2 (A) | Model 1 (linear trend) showing four-way interaction between T/V distinction, Language, Gender and Age.

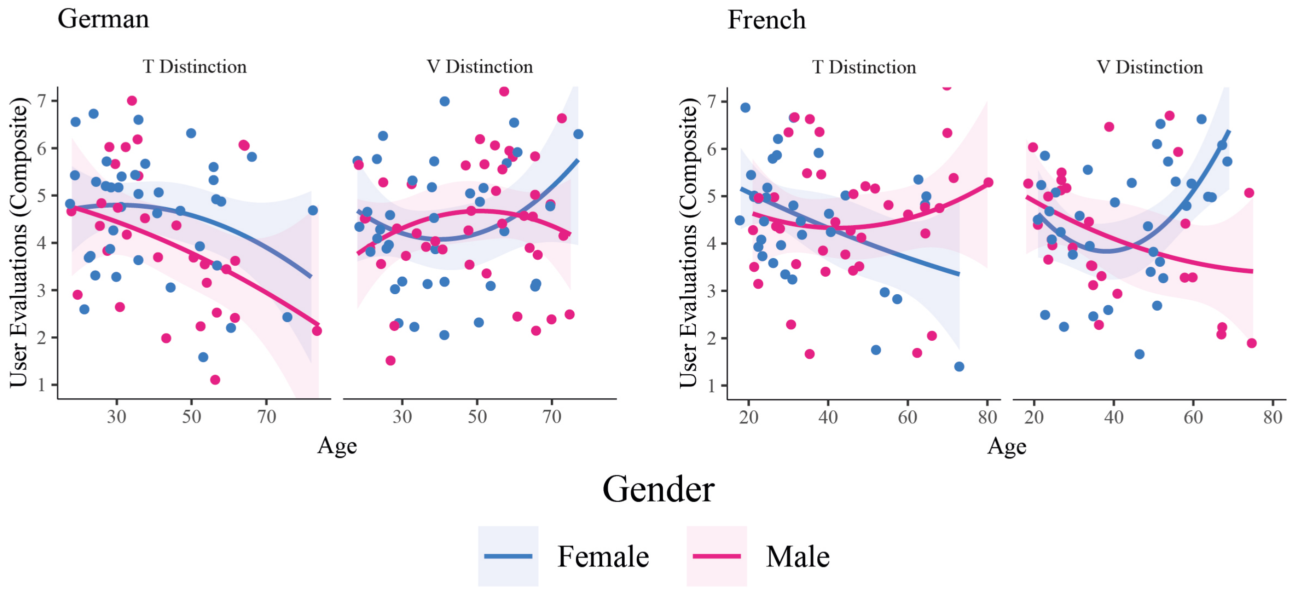

Figure 2 (B) | Model 2 (curvilinear trend) showing four-way interaction between T/V distinction, Language, Gender and Age.

4 Participant Demographic Profile

Further details on the demographic profile of participants can be found in Table 1. This details the number of participants by their gender, education level and mean group age, in each experimental condition (T/V distinction, Language).

**T/V Distinction**	**Language**	**Gender**	**Education**	***n***	**Age**	
					M	***SD***
**T form**	**French**	Women	Elementary School	1	65	-
			High School	19	32	14.36
			University	12	39	15.01
			Other	2	23	1.41
		Men	Elementary School	-	-	-
			High School	19	46	18.3
			University	19	44	14.85
			Other	-	-	-
	**German**	Women	Elementary School	2	49	10.61
			High School	30	40	17.44
			University	6	31	8.8
			Other	1	58	-
		Men	Elementary School	2	27	12.73
			High School	16	44	17.88
			University	11	46	13.28
			Other	1	33	-
**V form**	**French**	Women	Elementary School	1	60	-
			High School	19	44	12.68
			University	12	39	15.83
			Other	3	52	25.24
		Men	Elementary School	-	-	-
			High School	18	39	18.73
			University	11	41	15.77
			Other	-	-	-
	**German**	Women	Elementary School	1	41	-
			High School	29	39	16.08
			University	5	51	17.11
			Other	2	31	14.14
		Men	Elementary School	1	51	-
			High School	30	47	17.47
			University	11	54	17.43
			Other	-	-	-

Table 1 | Participant demographic profile (Gender, Education and Age) shown by Experimental Setting (T/V Distinction, Language).

Supplementary Material References

1. Qiu L, Benbasat I. Evaluating anthropomorphic product recommendation agents: A social relationship perspective to designing information systems. *J Manag Inf Syst* (2008) **25**:145–182. doi:10.2753/MIS0742-1222250405

2. Gefen D, Straub D. Managing User Trust in B2C e-Services. *e-Service J* (2003) **2**:7–24. doi:10.2979/esj.2003.2.2.7

3. Fussell SR, Kiesler S, Setlock LD, Yew V. "How people anthropomorphize robots," in *HRI 2008 - Proceedings of the 3rd ACM/IEEE International Conference on Human-Robot Interaction: Living with Robots.* (New York, New York, USA: ACM Press), 145–152. doi:10.1145/1349822.1349842

4. Verhagen T, van Nes J, Feldberg F, van Dolen W. Virtual customer service agents: Using social presence and personalization to shape online service encounters. *J Comput Commun* (2014) **19**:529–545. doi:10.1111/jcc4.12066

5. Lu B, Fan W, Zhou M. Social presence, trust, and social commerce purchase intention: An empirical research. *Comput Human Behav* (2016) **56**:225–237. doi:10.1016/j.chb.2015.11.057

6. Nass C, Moon Y. Machines and mindlessness: Social responses to computers. *J Soc Issues* (2000) **56**:81–103. doi:10.1111/0022-4537.00153

7. Araujo T. Living up to the chatbot hype: The influence of anthropomorphic design cues and communicative agency framing on conversational agent and company perceptions. *Comput Human Behav* (2018) **85**:183–189. doi:10.1016/j.chb.2018.03.051

8. Lee S, Lee N, Sah YJ. Perceiving a Mind in a Chatbot: Effect of Mind Perception and Social Cues on Co-presence, Closeness, and Intention to Use. *Int J Hum Comput Interact* (2020) **36**:930–940. doi:10.1080/10447318.2019.1699748

9. Dang BN, Westbrook RA, Njue SM, Giordano TP. Building trust and rapport early in the new doctor-patient relationship: a longitudinal qualitative study. *BMC Med Educ* (2017) **17**:1–10. doi:10.1186/s12909-017-0868-5

10. Timmerman GM. A concept analysis of intimacy. *Issues Ment Health Nurs* (1991) **12**:19–30. doi:10.3109/01612849109058207

11. Ho A, Hancock J, Miner AS. Psychological, Relational, and Emotional Effects of Self-Disclosure After Conversations With a Chatbot. *J Commun* (2018) **68**:712–733. doi:10.1093/joc/jqy026

12. Petronio S. Communication Boundary Management: A Theoretical Model of Managing

Disclosure of Private Information Between Marital Couples. *Commun Theory* (1991) **1**:311–335. doi:10.1111/j.1468-2885.1991.tb00023.x

13. Bansal G, Zahedi FM, Gefen D. The role of privacy assurance mechanisms in building trust and the moderating role of privacy concern. in *European Journal of Information Systems* (Palgrave Macmillan Ltd.), 624–644. doi:10.1057/ejis.2014.41

14. Skjuve M, Følstad A, Fostervold KI, Brandtzaeg PB. My Chatbot Companion - a Study of Human-Chatbot Relationships. *Int J Hum Comput Stud* (2021) **149**:102601. doi:10.1016/j.ijhcs.2021.102601

15. Lee YC, Yamashita N, Huang Y, Fu W. "'I Hear You, I Feel You': Encouraging Deep Self-disclosure through a Chatbot," in *Conference on Human Factors in Computing Systems - Proceedings* (New York, NY, USA: ACM), 1–12. doi:10.1145/3313831.3376175

16. Wu LW, Lii Y shuh, Wang CY. Managing innovation through co-production in interfirm partnering. *J Bus Res* (2015) **68**:2248–2253. doi:10.1016/j.jbusres.2015.06.006

17. Risling T, Martinez J, Young J, Thorp-Froslie N. Evaluating Patient Empowerment in Association With eHealth Technology: Scoping Review. *J Med Internet Res* (2017) **19**:e329. doi:10.2196/jmir.7809

18. Davis FD. Perceived Usefulness, Perceived Ease of Use, and User Acceptance of Information Technology. *Mis Q* (1989) **13**:319–340. doi:10.2307/249008

19. Zarouali B, Van Den Broeck E, Walrave M, Poels K. Predicting Consumer Responses to a Chatbot on Facebook. *Cyberpsychology, Behav Soc Netw* (2018) **21**:491–497. doi:10.1089/cyber.2017.0518

20. Rese A, Ganster L, Baier D. Chatbots in retailers' customer communication: How to measure their acceptance? *J Retail Consum Serv* (2020) **56**:102176. doi:10.1016/j.jretconser.2020.102176

21. Ashfaq M, Yun J, Yu S, Loureiro SMC. I, Chatbot: Modeling the determinants of users' satisfaction and continuance intention of AI-powered service agents. *Telemat Informatics* (2020) **54**:101473. doi:10.1016/j.tele.2020.101473

22. Diederich S, Brendel AB, Kolbe LM. Designing Anthropomorphic Enterprise Conversational Agents. *Bus Inf Syst Eng* (2020) **62**:193–209. doi:10.1007/s12599-020-00639-y

23. Bickmore T, Vardoulakis L, Jack B, Paasche-Orlow M. "Automated promotion of technology acceptance by clinicians using relational agents," in *Lecture Notes in Computer Science (including subseries Lecture Notes in Artificial Intelligence and Lecture Notes in Bioinformatics)* (Springer, Berlin, Heidelberg), 68–78. doi:10.1007/978-3-642-40415-3_6

24. Venkatesh V, Davis FD. A Theoretical Extension of the Technology Acceptance Model: Four Longitudinal Field Studies. *Manage Sci* (2000) **46**:186–204. doi:10.1287/mnsc.46.2.186.11926

25. Reichheld, F. F, Covey, S. R, Mekonnen A. The Ultimate Question: Driving Good Profits and True Growth. *J Targeting, Meas Anal Mark* (2006) **14**:369–370. doi:10.1057/palgrave.jt.5740195

26. Berman MA, Guthrie NL, Edwards KL, Appelbaum KJ, Njike VY, Eisenberg DM, Katz DL. Change in glycemic control with use of a digital therapeutic in adults with type 2 diabetes: Cohort study. *JMIR Diabetes* (2018) **3**:e4. doi:10.2196/diabetes.9591

27. Hauser-Ulrich S, Künzli H, Meier-Peterhans D, Kowatsch T. A Smartphone-Based Health Care Chatbot to Promote Self-Management of Chronic Pain (SELMA): Pilot Randomized Controlled Trial. *JMIR mHealth uHealth* (2020) **8**:1–23. doi:10.2196/15806

28. Go E, Sundar SS. Humanizing chatbots: The effects of visual, identity and conversational cues on humanness perceptions. *Comput Human Behav* (2019) **97**:304–316. doi:10.1016/j.chb.2019.01.020

29. Gupta A Sen. "Generalized Variance," in *Wiley StatsRef: Statistics Reference Online* doi:10.1002/9781118445112.stat01987
